# Advancement in Graphene-Based Materials and Their Nacre Inspired Composites for Armour Applications—A Review

**DOI:** 10.3390/nano11051239

**Published:** 2021-05-08

**Authors:** Jesuarockiam Naveen, Mohammad Jawaid, Kheng Lim Goh, Degalhal Mallikarjuna Reddy, Chandrasekar Muthukumar, Tamil Moli Loganathan, Koduri Naga Ganapathy Lakshmi Reshwanth

**Affiliations:** 1 School of Mechanical Engineering, Vellore Institute of Technology, Vellore 632014, India; gandhi.naveen66@gmail.com (J.N.); dmreddy@vit.ac.in (D.M.R.); reshwanthkoduri@gmail.com (K.N.G.L.R.); 2 Laboratory of Biocomposite Technology, Institute of Tropical Forestry and Forest Products (INTROP), Universiti Putra Malaysia, Serdang 43400, Malaysia; 3 Newcastle Research & Innovation Institute (NewRIIS), Singapore 609607, Singapore; kheng-lim.goh@newcastle.ac.uk; 4 Faculty of Science, Agriculture and Engineering, Newcastle University, Newcastle upon Tyne NE1 7RU, UK; 5 School of Aeronautical Sciences, Hindustan Institute of Technology & Science, Chennai 603103, India; chandrasekar.25j@gmail.com; 6 Department of Aerospace Engineering, Faculty of Engineering, Universiti Putra Malaysia, Serdang 43400, Malaysia; tamilmoli@yahoo.com

**Keywords:** body armour, graphene, artificial nacre, specific penetration energy, toughness, tensile strength

## Abstract

The development of armour systems with higher ballistic resistance and light weight has gained considerable attention as an increasing number of countries are recognising the need to build up advanced self-defence system to deter potential military conflicts and threats. Graphene is a two dimensional one-atom thick nanomaterial which possesses excellent tensile strength (130 GPa) and specific penetration energy (10 times higher than steel). It is also lightweight, tough and stiff and is expected to replace the current aramid fibre-based polymer composites. Currently, insights derived from the study of the nacre (natural armour system) are finding applications on the development of artificial nacre structures using graphene-based materials that can achieve high toughness and energy dissipation. The aim of this review is to discuss the potential of graphene-based nanomaterials with regard to the penetration energy, toughness and ballistic limit for personal body armour applications. This review addresses the cutting-edge research in the ballistic performance of graphene-based materials through theoretical, experimentation as well as simulations. The influence of fabrication techniques and interfacial interactions of graphene-based bioinspired polymer composites for ballistic application are also discussed. This review also covers the artificial nacre which is shown to exhibit superior mechanical and toughness behaviours.

## 1. Introduction

Ballistic impact produces shock waves which may induce severe trauma injuries to the soldiers. Aramid fibre-based soft and hard armours can absorb the impact energy of a projectile efficiently [1]. The current and future demand is creating a strong protection system against improvised explosive devices, multiple bullet strike and lethal ammunition [2]. In this point of view most of the bullet proof researchers, are aiming to manufacture a body armour which is stronger, flexible and light in weight. In 2013 the ballistic protection market in the globe reached $7.91 b and the expected growth rate is 42% during 2020.Global market of personal body armour alone may reach $3.1 b by 2027 [3]. High performance aramid fibres which are stronger, flexible and light in weight have been reinforced with polymers for body armour applications [3]. Currently, plant fibres and cellulose reinforcement plays an important role in developing sustainable and light weight composites [4,5,6]. These plant fibres were hybridized with aramid fibres to produce sustainable composites for body amour [7]. High performance epoxy matrices have been used as a matrix in the Kevlar-based polymer composites. In order to fabricate 100% biodegradable composites researchers are focusing on bio polymers which are biodegradable [8,9,10]. Further enhancing the energy absorption and to achieving higher energy dissipation, nano fillers are incorporated in the polymer matrix such as graphene-based fillers, carbon nanotube (CNT) etc. These nanomaterials had higher strength, stiffness, light weight, higher energy absorption and resistance to fracture makes them a most promising and potential materials for ballistic applications [11,12,13,14,15,16,17]. Graphene is one of the strongest nanomaterials [18]. Graphene-based materials are widely used in many applications such as automotive, aircraft, ballistic protection and electrochemical sensors/biosensors [19,20]. The strength and modulus of monolayer graphene is 125 GPa and 1 TPa respectively. It is a 2D isotropic one atom thick sheet with a thickness of approximately 0.335 nm [21].

Wetzel et al. found that graphene could improve the ballistic resistance of the body armour with light weight (100 times lighter than the current fibre reinforced polymer composite ballistic barriers) [22]. Moreover, the tensile wave speed of graphene is around 21.3 km/s which is higher than the wave speed of diamond (17.5 km/s). These lead to higher penetration resistance and faster spreading rate of impact energy [23]. The failure strain of graphene (0.25–0.30) is much higher than the conventional fibril armours (0.04). These superior properties of graphene and graphene-based nanocomposites significantly enhance the ballistic resistance and energy absorption compared to the aramid fibre-based composites. Currently, researchers have taken inspiration from the nacre (natural armour system) to fabricate the artificial nacre structure using graphene-based materials to obtain greater toughness and energy dissipation.

This review will focus on the cutting-edge research on the ballistic performance of graphene-based materials through theoretical, experimentation as well as numerical simulations. In addition, the effect of fabrication techniques and interfacial interactions of graphene-based nacre-inspired polymer composites for ballistic application have been discussed in detail.

## 2. Dynamic Mechanical Behaviours of Multilayer Graphene Sheets

**Figure 1:**
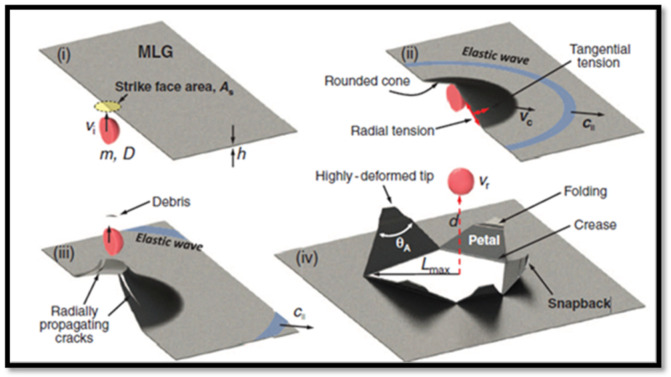
Penetration process (i) initial stage; (ii) cone formation stage; (iii) fracture stage; and (iv) post penetration stage, MLG: Multilayer graphene, V_i_ (impact speed), V_c_ (radial speed) V_r_ (residual speed) Reprinted with permission from AAAS [24]. Copyright 2014 AAAS.

Currently, graphene-based materials are replacing the existing aramid fibre-based body armour. Multilayer graphene (MLG) is an exceptional anisotropic material due to its layered arrangement composed of two-dimensional carbon lattices. Lee et al. [24] tested the MLG with microscopic projectile at extreme dynamic condition over a thickness range of 10–100 nm and found a higher strain rate of 10^7^/s. It has been observed that MLG possesses higher specific penetration energy (10 times higher than Steel at 600 m/s). This is mainly attributed to formation of cone waves and rapid propagation in the radial direction in the graphene layers. The penetration process is illustrated in Figure 1. During penetration process a micro bullet with mass (m) and impact speed (V_i_) impacts the strike face area (A_s_) of multilayer graphene (MLG). An elastic wave propagates radially at C_II_ followed by a conic deformation with radial speed Vc (Stage ii). Three to six cracks are initiated at the centre of A_s_ and propagates in radially outward direction resulting in the formation of the same number of petals (Stage iii). Moreover, the transferred momentum to MLG induces creasing and folding of each triangular-shaped petal while the elastic extension of the membrane is rapidly relaxed along the radial direction (Stage iv). Moreover, MLG dissipates the kinetic energy away from the impact zone. It has been suggested that the crack propagation in the polymeric composites can be prevented by incorporating multilayer graphene. Moreover, bio-inspired fabrication techniques enhance the toughness [25].

Kichul et al. have simulated the ballistic testing using silica and nickel projectiles over the graphene at supersonic initial velocity [26]. They have analysed the effect of supersonic impact on the graphene using RFF (reactive force field) method which describes the entire system. During penetration they have found the formation of pentagon/heptagon pairs at the crack edges. The calculated specific penetration energy was good in agreement with the experimental results reported by Lee at al. [24]. Zhaoxu et al. predicted the critical graphene membrane size with respect to the projectile size using analytical relationship verified by simulation data. Moreover, they have investigated the particular projectile size effect because of the reflection of the cone wave. The critical size relationship provides guidelines for the future microscale ballistic testing using 2D nanomaterials [27].

Zhaoxu et al. simulated the impact testing over a graphene plate using molecular dynamic simulation. It has been found that cylindrical projectiles penetrate the graphene plate at a lower velocity compared to spherical projectiles. Spalling like failure was observed in which the bottom section undergoes a wave superposition induced failure. Moreover, they have proposed a relationship to analyse the resisting pressure of graphitic plate during ballistic impact [28].

## 3. Predicted Strain Energy Density and Ballistic Limit Velocity of Graphene with Other Armour Materials

Wetzel et al. theoretically predicted the ballistic properties of graphene. Table 1 compares the predicted strain energy density and V50/V50 Kevlar ratio of widely used armour materials with graphene. The results have proven that the strain energy density of graphene is much higher than other materials. Additionally, when compared to Kevlar fibre, which is widely used as a ballistic material, graphene possesses an extraordinary strain energy density. Ballistic limit or limit velocity is the minimum velocity required to penetrate into the material. V50 represent the ballistic limit of individual material where as V50 Kevlar is the limit velocity of Kevlar. The V50/V50 Kevlar ratio of Kevlar is 1 and now it can be compared with other armour materials. CNT yarns had more acceptable ballistic limit than Kevlar, but the ballistic limit of graphene is much higher than CNT yarn as well as Kevlar. From these results it is evident that the graphene can potentially replace the existing body armour materials.

## 4. Nacre (Natural Armour System)-Like Graphene Structures

An interesting behaviour of graphene is that it is a planar 2D isotropic material with a capability to assemble in a perfect manner (multi-layer parallel arrangement). This extraordinary behaviour has encouraged engineers to fabricate the composite structures like nacre. Nacre is natural armour system used by the seashell to protect themselves from the external environment and it gives a physical protection [29]. Natural nacre contains a layer-by-layer structure of soft organic polymers and aragonite plates which makes it tougher and stronger. This layer-by-layer structure is more advantageous than others. The intercalation of polymeric matrix in the multilayer graphene significantly improves the mechanical behaviour [30,31]. Additionally, the unique brick-and-mortar structure of graphene within the multilayer graphene structure enhances the toughness and energy dissipation because of its self-healing van Der Waals interface [32].

### 4.1. Molecular Dynamic Simulation of Multi-Layer Graphene Based Polymer Composites

Wenjie et al. took inspiration from the natural nacre structure and they have proposed a molecular scale design with multilayer graphene/PMMA (poly methyl methacrylate) composites. They have performed a coarse-grained molecular dynamic to evaluate the interfacial failure mechanisms through pull-out simulations. From the simulation results they have concluded that the toughness and energy dissipation is significantly affected by the pull out at the graphene/PMMA interface and the yielding at the graphene layers. Beyond the critical point the yielding mode of failure turned into pull out failure. This modelling technique provides a platform to design the graphene-based polymer composites with optimum mechanical properties and toughness [33].

**Figure 2:**
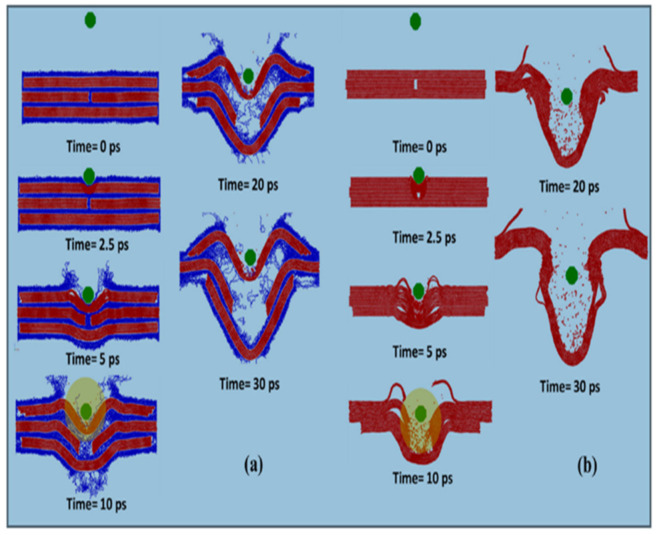
Snap shots during impact simulation (**a**) with polyethylene, (**b**) without polyethylene [34].

Liu et al. carried out a coarse grained molecular dynamic simulation to study the mechanical and energy absorption of multilayer graphene/polyethylene composites under spall loading condition. The energy absorption capability of the model increases with an increase of overlapping distance of graphene layers. Polymer matrix act as a protective shield to graphene during impact. From Figure 2a, it is clear that during impact the sample was bent, and the top polymer layer has been detached from the adjacent graphene layer. Rapid movement of polymeric chain leads to the formation of voids in the composites. Eventually, the bottom most graphene layer was detached from composite. Figure 2b shows the impact simulation without polymer, whereas the projectile penetrated into the top layer and breaks the sample into two parts. Hence the polyethylene polymer acts as a buffer. It decreases the contact force and protects the graphene layers. The obtained results showed that polymer matrix act as a cushion upon impact, which substantially decreases the maximum contact forces and thus inhibits the breakage of covalent bonds in the graphene flakes. Moreover, maximum contact force during the impact depends on the external surface area of impactors rather than the density of impactor [34].

### 4.2. Nacre-Like Multi-Layer Graphene Based Polymeric Composites

Masta et al. [35] studied the mechanical and ballistic performance of multilayer graphene (~35 vol.%)/polyvinyl alcohol (PVA) films. The 10 µm thick films were fabricated by using liquid exfoliation of graphene and filtration. The MLG/PVA composites exhibited high strength at lower strain rates compared to PVA. Nevertheless, the ductility was lower than PVA. Membrane stretching analysis was used to predict the ballistic limit of graphene/PVA composites comprising aligned large flakes. They have concluded that, these composites if manufacturable, will have higher ballistic limit (three times) compared to the best high-performance commercial composites with only 10 vol.% graphene reinforcement in PVA.

### 4.3. Nacre-Like Graphene Oxide Paper

Dikin et al. fabricated a graphene oxide paper using flow directed assembly of graphene oxide nano sheets. Interlocking feature of graphene oxide nano sheets provides an excellent macroscopic stiffness and flexibility. The strength and modulus of the graphene oxide paper is 133 MPa and 38 GPa respectively [36].

### 4.4. Nacre-Like Multi-Layer Graphene Oxide Based Polymeric Composites

Putz et al. fabricated a layer by layer homogeneous and highly ordered graphene oxide/polyvinyl alcohol composites with 50% graphene oxide content through vacuum assisted self-assembly technique. Compared to pure polymer nacre like nano composites film exhibited higher modulus [37]. Tan et al. fabricated the artificial nacre-like graphene oxide films through gel film transformation method. Different polymers (polyvinyl alcohol, polyethylene oxide and polyethylenimine) were blended into the aqueous graphene oxide solution. The interaction between the graphene oxide sheets can be modulated while blending the polymer. Electrostatic repulsive force of graphene sheets can be avoided through the attractive force in between the polymer and graphene sheets, and it promotes the gelation of graphene oxide. The resultant hydrogel (graphene oxide) after cast drying contains only 1–20 wt.% of polymer which makes the structure similar to nacre (layer by layer arrangement). The resulted tensile strength and the failure strain were 200 MPa and 3.0% respectively. It has been concluded that gel film transformation technique is a potential method to produce nacre like graphene oxide films in large scale with high strength and toughness [38].

### 4.5. Artificial Nacre with Alumina/Graphene Oxide/Poly (Vinyl Alcohol)

**Figure 3:**
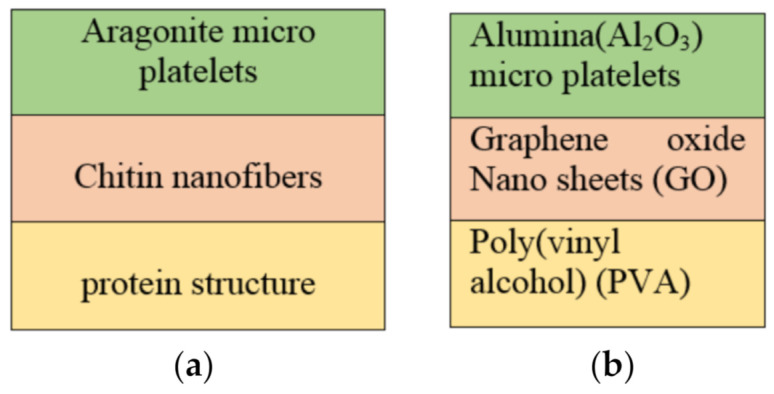
Layering sequence of (**a**) natural nacre vs (**b**) artificial nacre.

Naturally available nacre had an extraordinary strength and toughness due to its hierarchical arrangement of micro and nanostructures. This structure inspired the researchers to make high performance artificial composites using the same nacre design model. Figure 3a,b compares the arrangement of natural and the artificial-nacre structures. The bottom protein structure and the top micro platelets can be replaced with different polymeric matrices and metallic microplatelets respectively. The middle chitin nanofibres can be replaced with different multifunctional nanofillers.

Wang et al. developed artificial nacre through layer by layer technique. In this artificial nacre Al_2_O_3_ act as a brick and GO/PVA act as a mortar. The authors reported that even though artificial nacre composite structure possesses excellent strength, it exhibited moderate toughness. When the polymers are confined in between the graphene oxide nano platelets the structure will have superior properties, but it will lose its flexibility and ductile nature. Moreover, alumina microplatelets enhance the strength of the structure and it holds the ability of polymer matrix to deform and promotes the crack deflection. Artificial nacre structure showed an extraordinary strength and toughness (143 ± 13 MPa and 9.2 ± 2.7 MJ/m^3^), compared to natural nacre structures (80−135 MPa and 1.8 MJ/m^3^) [39].

### 4.6. Artificial Nacre with MoS_2_/rGO/TPU

Bertolazzi et al. developed an artificial nacre using MoS2/rGO/TPU. The breaking tensile strength and modulus of MoS2 is extremely higher (23 GPa and 270 GPa) for one monolayer and it has 2D nano sheet structure. This artificial nacre has been made through vacuum-assisted filtration self-assembly method. From the results it is understood that the tensile strength is 1.7 times greater than the natural nacre whereas the toughness is 3.8 times higher than the real nacre [40].

### 4.7. Artificial Nacre with rGO-DWCNTs-PVA Nanocomposites

Gong et al. [41] developed an artificial nacre using 2D rGO (reduced graphene oxide) nanosheets and 1D double-walled carbon nanotubes (DWNTs) with polyvinyl alcohol (PVA). It exhibits excellent fatigue resistance and can energy absorption characteristics. This research has opened new avenues for creating nacre inspired structures with different nano fillers even with 1D building blocks (cellulose nano fibre) and double walled carbon nanotube or 2-dimensional building blocks like montmorillonite (MMT). Additionally, it has been proven that combining two nanomaterials like graphene and CNT enhances the mechanical and energy absorption of polymeric composites [42].

### 4.8. Graphene-Based Bio-Inspired Polymer Nano Composites—Fabrication Techniques

Nacre exhibits excellent mechanical toughness due to the hierarchical micro/nano structure and better interfacial interactions [43,44]. This nacre provides a golden template to fabricate bio inspired artificial nacre like composite structure [45]. Several preparation methodologies were utilized by the researchers to fabricate the artificial nacre or bio inspired polymer nano composites such as layer-by-layer [46], evaporation [47], filtration [48], freeze casting [49], hydrogel casting and electrophoretic deposition [50]. These methods ensure a homogeneous dispersion, perfect alignment and great interfacial interactions in the bio inspired polymer nano composites [51]. These advanced techniques will be discussed in detail in the following sections.

#### 4.8.1. Layer-by-Layer Fabrication Technique

**Figure 4:**
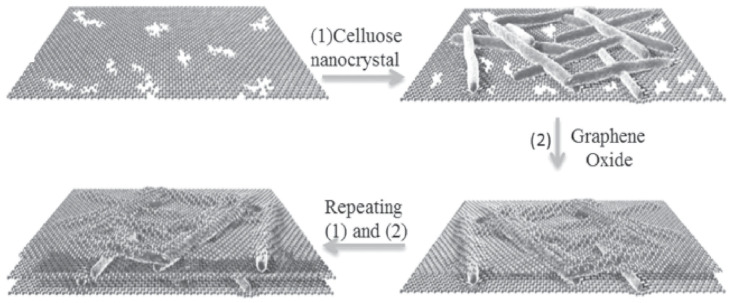
Preparation of the laminated cellulose nanocrystals/graphene oxide nanomembranes. Reproduced with permission from [53]. Copyright 2015 John Wiley and Sons.

Decher et al. proposed the layer-by-layer fabrication technique for multilayer films based on electrostatic attraction mechanism. This could be achieved by alternative deposition of polyelectrolytes with opposite charges [51,52].The process of this fabrication technique is described as follows: (i) Initial cleaning and surface treatment of substrate; (ii) Deposition and absorption of first layer film on the substrate; (iii) Cleaning of substrate with first layer and washing thoroughly before the second layer absorption which protects the structure from pollution and maintains the stability; (iv) Submerging the substrate into the second solution to form the second layer over the first layer based on the driving force; (v) Cleaning the substrate with the first and second layers and washing again; (vi) Repeat the process ii, iii, iv, and v. Xiong et al. fabricated a high performance bio-inspired nano composites with layer by layer technique (Figure 4) using GO-cellulose nanocrystals and reported the strength and modulus are 655 MPa and 169 GPa respectively [53]. Presence of high concentration of surface anionic functional groups improves the effective “gluing” of CNCs to primed GO sheets via noncovalent, strong ionic interactions, and hydrogen bonding. Overall, this approach has some advantages and limitation. The main advantage of this layer-by-layer fabrication technique is precise control of hierarchical structure which provides superior mechanical and energy absorption properties. On the other hand, it is a time-consuming fabrication process.

#### 4.8.2. Evaporation

**Figure 5:**
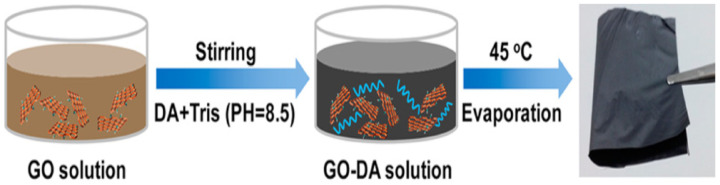
Fabrication of artificial nacre using graphene oxide /dopolyamine (DA). Reproduced with permission from [54]. Further permissions related to the material excerpted should be directed to the ACS. Copyright 2014 ACS.

Compared to layer-by-layer fabrication technique, evaporation-based fabrication technique is a very simple procedure to produce bio-inspired nanocomposites. The nano sheets in the solution could form a perfectly aligned low energy structure after evaporation of solvent. Cui et al. fabricated the bio inspired graphene oxide/dopolyamine (DA) nanocomposites through evaporation technique as shown in Figure 5 [54]. Tensile strength and toughness of graphene oxide/polyamine nanocomposites were investigated. From the results it was observed that the toughness and tensile strength of the nanocomposites are 2 and 1.5 times greater than the natural nacre. This is attributed to the strong covalent bond between the graphene oxide and polyamine. Moreover, presence of oxygen functionalities in graphene oxide enhanced the dispersion in different matrices. Though the higher temperature could accelerate the evaporation, it is not conducive to the formation of an orderly structure. Similar to the layer-by-layer technique, this evaporation approach is also a time-consuming process. Accelerating the evaporation process could reduce the mechanical strength and energy absorption of the bio-inspired nanocomposites.

#### 4.8.3. Filtration

Filtration is also a simple and efficient method to fabricate bio-inspired nanocomposites [55,56]. The nano sheets were initially immersed in the solvent. This mixture was poured over the filter assisted with vacuum. An ordered bio-inspired structure was formulated with the flow of solvent. Wan et al. [25] fabricated the reduced graphene oxide-polyacrylic acid nano composites through vacuum assisted filtration technique and achieved greater tensile strength and toughness. Further they have evaluated the effect of relative humidity on the tensile and toughness behaviour of nano composites. This vacuum filtration technique was utilized by many researchers to fabricate high performance bio-inspired nano structures [43]. The drawbacks of using this filtration technique are size and filtration speed. Due to the size restriction, it is not possible to scale up the bio-inspired nanocomposites. Additionally, the filtration process takes more time. Hence, similar to other layer-by-layer and evaporation techniques, it is also a time-consuming fabrication technique.

#### 4.8.4. Freeze Casting

Generally, sea water contains impurities, dust particles, micro-organisms etc. However, in ice the impurities were expelled into the interstices of the ice [57,58]. Based on this phenomenon, a novel freeze casting technique has been developed in 2006. This moulding technique is used to make complex bulk structures. Freeze casting is mainly used for fabricating porous ceramic materials [59]. It is a novel in expensive and eco-friendly fabrication technique to make bulk bio inspired nano composites. The artificial nacre like structure made up of freeze casting has shown higher toughness, high tensile strength and fracture toughness compared to natural nacre. Recently researchers have developed a bidirectional freezing technique to scale up the structure [60]. Even though this freeze casted structure exhibited superior mechanical properties and toughness, precise nacre like structure could not be replicated by using freeze casting technique.

#### 4.8.5. Hydrogel Casting

**Figure 6:**
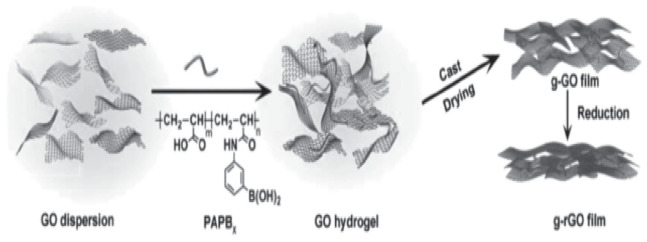
Preparation of graphene oxide film. Reproduced with permission from [64]. Copyright 2014 John Wiley and Sons.

Hydrogel casting is a techno economical approach to produce bio-inspired composites. This technique is widely used to fabricate graphene-based bio-inspired polymer nanocomposites [61,62]. Due to the hydrophobic and hydrophilic groups on the surface of the graphene oxide nano sheets, it can swell in water and easily assemble into three-dimensional network structures. It is essential to introduce crosslinking agent while preparing graphene oxide hydrogel in order to fabricate the bio-inspired nanocomposite structure through hydrogel casting. Further, graphene oxide-based hydrogels show an excellent reversibility and lower critical gelation concentration [61]. The interaction between the graphene oxide nano sheets have made gelation of graphene oxide which includes electrostatic interaction, hydrogen bonding, coordination and π-π stacking [61,62]. It is an efficient and simple method to produce large scale hierarchical bio-inspired nano structure through different interfacial interactions [61,63]. Zhang et al. have developed graphene oxide/poly (acrylic acid-co-(4-acrylamidophenyl) boronic acid) (PAPBx) nanocomposites. The critical gelation concentration of PAPBx is less than 1 wt.% which facilitates the graphene oxide to form hydrogel. Formation of homogeneous bio inspired nano composite has shown improvement in mechanical properties. The GO and rGO composite films made by gel-film transformation technique illustrated in Figure 6 were named g-GO and g-rGO films, respectively. Compared to pure graphene oxide film, PAPBx based nanocomposites exhibited higher mechanical properties. However, the major drawback of this approach is precise control of bio-inspired laminated structure, which is a challenging task.

#### 4.8.6. Electrophoretic Deposition

This technique has attracted the researchers because of its enormous advantages than other approaches. The cost of fabrication of artificial nacre through this approach is cheaper. Further it is the fastest fabrication technique among the artificial nacre fabrication techniques. Precise control of the hierarchical structure could be achieved using this approach. However, currently, researchers are using this technique for preparing thin bio-inspired polymer nano composites with a well laminated hierarchical structure [65]. The electrophoretic deposition process involves two process steps. Initially, the electrodes attract the oppositely charged particles inside an electric field. Then the particles are deposited on the surface of the electrodes and form a thin film. The drawback of this electrophoretic deposition approach is fabrication of thick artificial nacre structure is not possible because of its limitation in the setup [51]. The Table 2 compares the merits and limitations of nacre inspired manufacturing technologies.

## 5. 3D Graphene Materials

Graphene aerogel is one of the emerging 3D graphene material which is used to fabricate polymer composites. Wang et al. [66] developed this graphene aerogel-based epoxy composites. Initially, conventional modified hummer’s method was employed to produce dispersed graphene oxide using natural graphite flakes. Then the dispersed graphene oxide was diluted (by using DI water) and sonicated for 30 min. Graphene hydrogel (GH) has been fabricated using hydroiodic acid as a reducing agent through chemical reduction and self-assembly of graphene oxide sheets.

The dispersed graphene oxide and hydroiodic acid were mixed in a magnetic stirrer for several minutes. The final mixture was then kept in an autoclave for 24 h at 120°. The mixture (GH) was cooled at room temperature and cleaned using distilled water to remove the impurities completely. The obtained GH was kept at −30° (for 12 h) and freeze-dried at −80° (for 48 h) in a freeze drier to produce graphene aerogel. Eventually the graphene aerogel/epoxy matrix composites were prepared through vacuum assisted infiltration method. They have concluded that graphene aerogel drastically improves the mechanical strength, energy dissipation and fracture toughness of epoxy composites. Moreover, graphene aerogel enhances the crack propagation resistance of the GA/epoxy composites [66].

## 6. Effect of Interfacial Interactions

The material response to mechanical loads and the energy absorption characteristics of the bio-inspired nanocomposites not only rely on the inherent characteristics of the constituents but also depends on the sequence of assembling building blocks and the interfacial adhesion [67]. Even though the nano fillers exhibit superior properties when added to the composites, aggregation of nano particles at higher weight % as well as poor wetting with resin in the polymer matrix could result in lower performance of the composite structure under various loading scenarios and elevated temperature conditions. Numerous methodologies were introduced to solve these limitations, to strengthen and enhance the interfacial bonding between the constituents of the bio-based nanocomposites. This could be achieved by various mechanisms as follows: (a) covalent bonding and (b) non-covalent bonding (hydrogen bonding, ionic bonding, and p–p interactions). Even though the non-covalent bonding had higher mechanical and energy absorption behaviour they cannot withstand harsh situations or sudden mechanical loading. Covalent bonding has shown strong interaction in between the constituents of the polymer composites. It can be employed to fabricate high performance polymer composites [68]. This strong interaction can effectively transfer the load and maintain the integrity of the structure. Due to this, weak interactions which induce crack deflection and plastic deformation could be destroyed. This strategy is very useful while fabricating tough and stiff polymer-based nanocomposites.

### 6.1. Non-Covalent Bonding

The interfacial interaction is inevitable for achieving superior properties of the bio inspired polymer composites. Non-covalent bonding between the building blocks of artificial nacre can enhance the mechanical properties and energy absorption while the covalent bonds could improve the strength, toughness, strain resistance and fatigue strength of the structure. In the following section, different types of non-covalent bonding will be discussed in detail.

#### 6.1.1. Hydrogen Bonding

Hydrogen bonding is quite common at the intermolecular or intra-molecular level [69]. Even though hydrogen bonding is weaker in nature; it significantly affects the molecular physical structure of the material. Hydrogen bonding is ubiquitous in artificial nacre structure due to the presence of large amount of the oxygen functional groups on the surface of the graphene oxide. Dikin et al. fabricated graphene oxide film by filtration technique. Hydrogen bonding between the graphene oxide and H2O was found to enhance the mechanical properties for the GO film [36]. Incorporation of 4.51 wt.% PAA with rGO resulted in nanocomposite film with the strength and toughness, twice and thrice greater than the pure GO film [25].

#### 6.1.2. Ionic Bonding

Addition of a small quantity of the metal ion into the biomaterial enhances the mechanical properties by forming ionic bonds with protein structure of the biomaterial. Abundantly available oxygen functional groups in the graphene oxide (GO) nanosheets are favourable to improve the coordination with metal ions. Park et al. prepared the GO-based films with Mg^2+^ and Ca^2+^ at less than 1 wt.%. They found that the metal ion greatly enhances the mechanical performance of GO film by forming ionic bond with the GO nano sheets in the proximityAdditionally, they found that the GO-Mg^2+^ possess higher mechanical performance than GO-Ca^2+^ because of lower ionic radius of Mg^2+^ than Ca^2+^ [70].

#### 6.1.3. π-π Interaction

Strong π–π interaction in the graphene nano sheets improves the stability of the structure. Zhang et al. found that addition of less than 1 wt.% PAPBx with GO nano sheets produces hydrogel which enhances the π-π interaction with GO nano sheets as confirmed from the spectra pattern in the adsorption and fluorescence spectra analysis [71]. The tensile strength and toughness of GO-PAPBx structure was 382 MPa and 7.5 MJ/m^3^ respectively [64].

### 6.2. Covalent Bonding

Even though the non-covalent bonding could improve the mechanical properties drastically, it is inactive in salt solutions [45]. Covalent bonding exhibited greater robustness in the artificial nacre structure. Covalent bonding in the artificial nacre structure can result in a 3D network using the linear molecule in the polymer as well as with the branched networks in the polymer which will be discussed in detail in the sections below:

#### 6.2.1. Covalent Bonding through the Linear Molecule in the Polymer

Linear molecule in the polymer can form a strong covalent bonding network with the GO nano sheets leading to a nanocomposite film with superior mechanical properties. Among the various linear molecules, borate and GA were commonly employed to improve the mechanical properties of GO films. Both the tensile strength and storage modulus increased by 25% and 266% with the addition of 0.94 wt.% of borate in the GO film [72]. Gao et al. fabricated GO-GA based artificial nacre structure and they found that addition of GA led to nanomaterial with superior tensile strength and young’s modulus than the GO film. However, toughness for the GO-GA nanocomposite declined due to the shorter GA chains [73]. In their work, Cheng et al. [74] showed that the toughness can be improved by employing long chain polymer namely 10, 12-pentacosadiyn-1-ol (PCDO) with GO.

#### 6.2.2. Branched Polymer

Multiple functional groups in the branched polymer compared to linear molecule helps in increased crosslinking between the polymer and GO such that significant rise in the mechanical properties can be achieved. The commonly available branched polymer to prepare artificial nacre is hyper branched polyglycerol (HPG). GO-HPG nanocomposite had the tensile strength of 642 MPa which is higher than pure GO film (555 MPa) [75]. It must be noted that the studies on GO based branched polymers is limited due to the difficulty in synthesizing the branched polymer with GO.

#### 6.2.3. 3D Network

Thermosetting polymers like epoxy have reactive functional groups that can be grafted on the surface of the building blocks of an artificial nacre and crosslinked with adjacent blocks to form a 3D network structure. Ming et al. fabricated the graphene foam (GF)/epoxy nano composites [76]. GF obtained from GO films was blended in the epoxy followed by fabrication using hot press moulding to form the GF/epoxy composites. Both the tensile strength and the modulus was found to be enhanced by 23% and 136% respectively.

Thus, it is clear that mechanical properties of the bio inspired nano composite structures can be augmented with the use of polymers which can form the covalent bonding mechanisms as discussed above. However, precise control of the interfacial interaction to achieve optimum performance under various mechanical loads is still a challenging task.

### 6.3. Conventional and Bioinspired Nano Composite Structure

**Figure 7:**
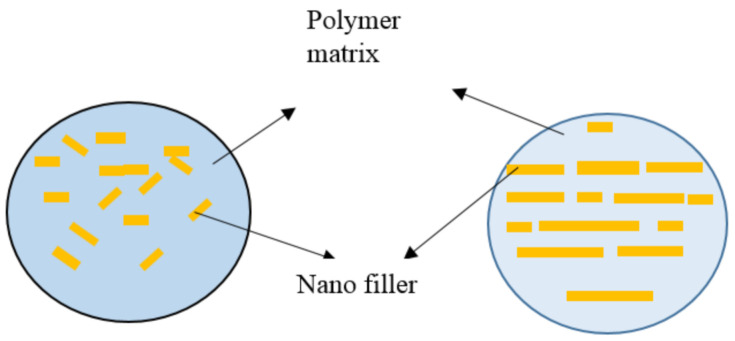
Conventional and bio-inspired nano composite structure.

Figure 7 compares the conventional polymeric composites and the bioinspired composites structures. The main advantage of bio inspired structure is the uniform dispersion of the nano filler in the polymer matrix throughout the structure and higher rate of the interfacial interaction which imparts superior mechanical properties and better energy absorption behaviour to the polymer. Ni et al. [77] developed a bioinspired structure which possess higher strength and toughness of 538.8 MPa and 16.1 MJ/m^3^ respectively. This could be achieved via cross linking of graphene nano sheets through π-π interactions by using a pyrene group conjugate molecule on both ends.

Shahzadi et al. [78] have fabricated nacre inspired nanocomposite material containing carboxymethyl cellulose (CMC) with GO and a combination of the reduced graphene oxide/alumina (rGO/Al). They reported improvement in strength due to the strong covalent bonding between rGO and alumina. Irrespective of the results, the synthesis process is relatively tedious and time consuming. They have concluded that strong covalent bonding drastically enhances the strength and toughness of the graphene based nano structures.

Uniform dispersion of nano filler into the matrix were achieved using techniques such as surface grafting and coupling reaction, polymer wrapping and surfactant adsorption [44]. Surface grafting is an effective way to modify the surface of the nano filler. Some functional groups were introduced on the surface of the nano fillers which strengthen the chemical covalent bonding with the polymer matrix. Researchers grafted the epoxy matrix with nano fillers after diazotization and were able to achieve uniform dispersion of nano fillers in the polymer [79]. Polymer wrapping approach functionalizes the nano fillers through van Der Waals and π–π stacking that could improve the interfacial interaction of the nano filler with the polymer. Surfactant adsorption is another technique that can be used to functionalize the surface of the nano fillers for better dispersion and wetting of the nano filler with the polymer matrix [80]. One such commonly used surfactant is polyoxyethylene [81].

Table 3 shows the energy absorption, ballistic limit, tensile strength and toughness of graphene-based bio-inspired composites. These properties are essential for an efficient armour. During ballistic impact the FRP composite is subjected to different failure modes such as shear plugging, matrix cracking, delamination and tensile failure on the read side of the ballistic panel. Energy absorption can be investigated through drop weight impact test or gas gun experimental set up. From the observation it was found that multilayer graphene/polypeptide composites exhibited excellent energy absorption. Nonetheless, energy absorption of graphene based polymeric composites with different manufacturing techniques has to be explored. Similarly, ballistic limit of graphene based polymeric composites with different manufacturing techniques has to be explored. On the other hand, excellent toughness is essential for an efficient armour system. From the analysis it was noticed that GO/polyurethane composites exhibited excellent toughness compared to other graphene based polymeric composites.

## 7. Conclusions and Future Recommendations

Due to increase in demand for flexible, light weight and robust body armour it is essential to find an alternative for the current aramid fibre-based protection system. Graphene is one of the strongest enormous energy absorbers, lightweight, tough and stiffest material which can replace the current aramid fibre-based material. Further the extraordinary self-aligning behaviour of graphene encouraged the material scientist to fabricate the composite structures like nacre. This behaviour could overcome the drawbacks of conventional fabrication technique. The most common problem while fabricating the nanocomposites is the non-uniform dispersion of nano particles in the matrix. Uniform dispersion could be achieved through surface grafting and coupling reaction, polymer wrapping and surfactant adsorption. Nacre inspired graphene-based system required carefully arranged hierarchical multi-layered structures to achieve excellent stiffness, strength, toughness, energy absorption, impact resistance and light weight, which has to be considered while designing high performance armour system [85]. Overall, this graphene-based materials act as a potential future body armours with highest energy absorption. Micro level experimentation and simulations proved that graphene is suitable for ballistic application. However, as per NIJ standards large scale ballistic panels should be tested for commercialization of graphene based soft, stiff and hard body armours. The armour panels should not fail until six shots without penetration as per NIJ standards. The ballistic performance of graphene-based materials can be improved with shear thickening fluids or modified shear thickening fluids. Blunt trauma testing of graphene-based materials has been explored to simulate the human body being penetrated by the projectile. On the other hand, non-biodegradable polymers can be replaced with bio-based polymers to enhance the biodegradability of the resulting armour panels.

## Figures and Tables

**Table 1:** Comparison of ballistic limit velocity ratio and strain energy density. Reprinted with permission from [22]. Copyright 2015 Elsevier.

Material	Strain Energy Density (J/g)	V50/V50 Kevlar
Kevlar129	38.7	1.00
Dyneema SK-76	48.1	1.13
Carbon fibre	26.8	0.99
CNT yarn	121	1.97
Aluminium alloy5083	9.5	0.76
Titanium alloyTi-6-4	29.4	1.10
Graphene	8350	11.6

V50—ballistic limit.

**Table 2:** Merits and limitations of nacre inspired manufacturing technologies.

Sl.No	Fabrication Technique	Merits	Limitations
1.	Layer by layer	Layered structure can be controlled precisely	Time consuming process.
2.	Evaporation	The evaporation procedure is quiet easy.	Precise control of the structure is difficult
3.	Filtration	Simple operating procedure.	Scaling up is a tedious and time consuming process
4.	Freeze casting	Suitable to fabricate bulk materials	Consumes more energy
5.	Hydrogel casting	Economical technique.	Controlling the layered structure is difficult.
6.	Electrophoretic deposition	Precise control of the structure	Fabrication of thick film is very difficult.

**Table 3:** Properties of graphene and nacre-inspired graphene-based composites for armour applications.

Material		Fabrication Techniques	Energy Absorption	Ballistic Limit	Stress (Tensile Strength)	Toughness	Ref.
Type of Graphene	Polymer						
Multilayer graphene	-	Overlapping	3 MJ	-	50 MPa	7 × 10^3^ MJ/m^3^	[32]
Multilayer graphene	Poly vinyl alcohol	liquid exfoliation	-	15 m/s	50 MPa	-	[35]
Graphene oxide paper	-	Flow-directed assembly of individual graphene oxide sheets	-	-	130 MPa	-	[36]
Graphene oxide	Poly Vinyl alcohol or hydrophobic poly(methyl methacrylate)	Filtration	-	-	102.9 MPa	-	[37]
Graphene oxide	Water soluble	Gel film Transformation (GFT)	-	-	200 MPa	8.98 ± 0.73 MJ/m^3^ (varies with different materials)	[38]
Graphene oxide	Poly vinyl alcohol	Layer by Layer	-	-	143 ± 13 MPa	9.2 ± 2.7 MJ/m^3^	[39]
Graphene oxide	Poly vinyl alcohol	Evaporation	-	-	240.4 ± 30.8 MPa	2.0 ± 0.5 MJ/m^3^	[43]
Graphene oxide	Al_2_O_3_ platelets and chitosan	Hydrogen bonding	-	-	152 MPa (varies on linkages)	75 MJ/m^3^ (varies on linkages)	[45]
Graphene	Poly vinyl alcohol	Layer-by-Layer	-	-	219 ± 19 MPa	-	[47]
Bioinspired Graphene	Bio polymer	Hydrogel Casting	-	-	382 MPa (varies while increase in bio polymers)	7.5 MJ/m^3^	[51]
		Layer-by-Layer	-	-	300 MPa (varies while increase in bio polymers)	75 MJ/m^3^ (varies with GO sheets)	
		Filtration	-	-	133 MPa (varies while increase in bio polymers)	-	
		Evaporation	-	-	-	-	
Graphene oxide	Fibre-based biopolymers and polymer nano composites.	Drop-casting or vacuum-assisted filtration	-	-	400 MPa (varies by linkages)	3.9 ± 0.5 MJ/m^3^ (varies on polymers)	[53]
Graphene oxide	Poly vinyl alcohol	Layer by Layer			91.2 ± 1.6 MPa (varies by linkages)	1.4 ± 0.1 MJ/m^3^ (varies on different sheet linkages)	[55]
Multilayered Graphene	Polypeptide	Filtration	6000 J	-	351 MPa (maximum)	-	[56]
Graphene oxide	Poly acrylic acid	Vacuum-assisted filtration	-	-	179.03 ± 4.55 MPa (Depends on humidity varies)	6.04 ± 0.49 MJ/m^3^ (Depends on humidity varies)	[25]
Graphene oxide	Bio inspired nano composite	Evaporation	-	-	374.1 ± 22.8 MPa (2.6 times increased than original)	9.2 ± 0.8 MJ/m^3^ (3.3 times increased than original)	[43]
Graphene oxide	Poly crystalline rings	Hydrogel casting	-	-	1.91 ± 0.08 MPa	-	[62]
Chemically modified graphene	Hydrophilic polymer	Hydrogel casting	-	-	-	-	[63]
Graphene oxide	poly (acrylic acid-co-acrylamidophenylboronic acid)	Gel Film Transformation technique	-	-	382 ± 12 MPa	7.50 ± 0.4 MJ/m^3^	[64]
3D Graphene	hydroxyapatite	Hydrothermal method	-	-		2.8 MPa·m^0.5^	[82]
Thermally reduced grapheme oxide	Natural polymer	Compression moulding	-	-	3.5 GPa	-	[83]
Graphene oxide	Polyurethane	In-situ polymerization			40.2 ± 1.8 MPa (varies with linkages)	192.9 ± 4.7 MJ/m^3^ (varies with linkages)	[84]

## Data Availability

Data sharing not applicable. No new data were created or analyzed in this study. Data sharing is not applicable to this article.
